# Local Structure of Ion Pair Interaction in Lapatinib Amorphous Dispersions characterized by Synchrotron X-Ray diffraction and Pair Distribution Function Analysis

**DOI:** 10.1038/srep46367

**Published:** 2017-04-11

**Authors:** Gabriel L. B. de Araujo, Chris J. Benmore, Stephen R. Byrn

**Affiliations:** 1 Department of Pharmacy, Faculty of Pharmaceutical Sciences, University of Sao Paulo, Sao Paulo, SP, 05508-900, Brazil; 2 Department of Industrial and Physical Pharmacy, Purdue University, West Lafayette, Indiana, 47906, United States; 3 X-ray Science Division, Advanced Photon Source, Argonne National Laboratory, Illinois, 60439, United States

## Abstract

For many years, the idea of analyzing atom-atom contacts in amorphous drug-polymer systems has been of major interest, because this method has always had the potential to differentiate between amorphous systems with domains and amorphous systems which are molecular mixtures. In this study, local structure of ionic and noninonic interactions were studied by High-Energy X-ray Diffraction and Pair Distribution Function (PDF) analysis in amorphous solid dispersions of lapatinib in hypromellose phthalate (HPMCP) and hypromellose (HPMC-E3). The strategy of extracting lapatinib intermolecular drug interactions from the total PDF x-ray pattern was successfully applied allowing the detection of distinct nearest neighbor contacts for the HPMC-E3 rich preparations showing that lapatinib molecules do not cluster in the same way as observed in HPMC-P, where ionic interactions are present. Orientational correlations up to nearest neighbor molecules at about 4.3 Å were observed for polymer rich samples; both observations showed strong correlation to the stability of the systems. Finally, the superior physical stability of 1:3 LP:HPMCP was consistent with the absence of significant intermolecular interactions in (∆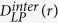) in the range of 3.0 to 6.0 Å, which are attributed to C-C, C-N and C-O nearest neighbor contacts present in drug-drug interactions.

The establishment of relationships between structural packing and physico-chemical properties has become fundamental for the modern pharmaceutical development^1^,^2^,^3^,^4^,^5^. In order to overcome poor solubility and improve bioavailability the use of amorphous and nanocrystalline systems are growing, as is the necessity of improved methods to characterize them^6^. In amorphous systems the atoms are ordered primarily at short (2–5 Å) and medium-range (5–20 Å.)^7^ distances. This makes atomic structure determination a challenging task that cannot be properly addressed by classical crystallography^8^,^9^,^10^,^11^. The quote of Alfred North Whitehead, highlighted by Mackey^8^ in his work about generalized crystallography fits well to better visualize this problem: *“A crystal lacks rhythm from excess of pattern, while a fog is unrhythmic in that it exhibits a patternless confusion of detail”*^12^. Although the presence of chemical bonds between molecules within the system ensures not all ordering is lost and some degree of preferred orientation can and often does persist in an amorphous solid.

Towards finding order within the chaos of amorphous systems the combination of total X-ray diffraction and pair distribution function analysis (PDF) has been utilized in the last decades as a valuable approach and it is being used more and more in the study of great variety of glasses^13^,^14^,^15^,^16^,^17^. The PDF expresses the probability of finding a pair of atoms which are separated from each other by a determinate distance (r), represented by peaks generated by intramolecular and intermolecular interactions, taking into account their abundance in the system^13^,^15^. Examples of common events observed in PDF patterns and information that can be extracted from them are summarized in Table 1.

Besides its wide use in the study of inorganic glasses^7^,^18^, the pharmaceutical literature is expanding in the applications of the technique^5^,^14^,^15^. Several groups have used conventional X-ray diffraction patterns to generate PDF patterns^19^,^20^,^21^,^22^. Due to inherent limitations of those low energy laboratory X-ray sources^11^ it is possible that these patterns contain artifacts, nevertheless, these papers illustrate the type of studies that can be done with PDF. Sheth and co-workers accessed the local structure of amorphous piroxicam prepared by cryogrinding from different polymorphs using this tool^22^. Differences in PDF patterns during grinding indicated the existence of an intermediary nanocrystalline phase during the amorphization process of crystalline form II, not observed for crystalline form I^10^,^22^. Additionally, medium-range similarity observed in PDF amorphous and crystalline phases seems to be indicative of residual memory that can drives recrystallization towards a specific polymorph^10^. Newman *et al*. reported the use of PDF to evaluate phase miscibility in indomethacin-PVP and trehalose–dextran mixtures^21^. In the first system, the PDF results indicated a complete miscibility with a good agreement with the number of glass transitions found; in the second system, the PDF indicated potential phase separation not detected by differential scanning calorimetry (DSC), suggesting that the system was a solid nanosuspension with nanometer sized amorphous domains lower than 30 nm.

The introduction of high-energy x-rays produced by synchrotron radiation has allowed the use of short wavelengths and access to higher Q-values (Qmáx ≥ 20 Å^−1^), a prerequisite for increased real space resolution and achieving more precise and accurate data^11^,^23^. Billinge and co-workers using a Q_máx_ = 20 Å^−1^ obtained a real-space resolution of 0.31 Å in the study of amorphous indomethacin and carbamazepine samples, being able to better identify differences in the molecular packing^11^. Benmore and colleagues used high-energy X-ray diffraction and neutron diffraction in combination with PDF to study vitrified carbamazepine, cinnarizine, miconazole, clotrimazole and probucol prepared by acoustic levitator^14^. The high resolution allowed the differentiation of significant intermolecular and intramolecular interactions in the range of 5–15 Å; for e.g. a shift in a peak was observed at 3.78 Å to 3.94 Å was identified due to changes in the orientation of the phenyl ring of clotrimazole caused by the vitrification process^14^.

In the present work we report the potential application of PDF analysis and high-energy X-ray diffraction as a tool to study acid-base interactions and drug-excipient interactions in amorphous systems. Acid-base interactions between drug-excipient play an important role in the physical stabilization of amorphous systems^24^,^25^,^26^. An important small molecule tyrosine kinase inhibitor used in breast cancer therapy, lapatinib (LP), has been chosen as model. It is a weak base with a secondary amine group (pKa = 7.26) and it was recently reported to form a strong intermolecular ionic interaction with acidic polymers^25^,^26^, promoting the formation of highly stable amorphous solid dispersions.

## Results and Discussion

**Figure 1:**
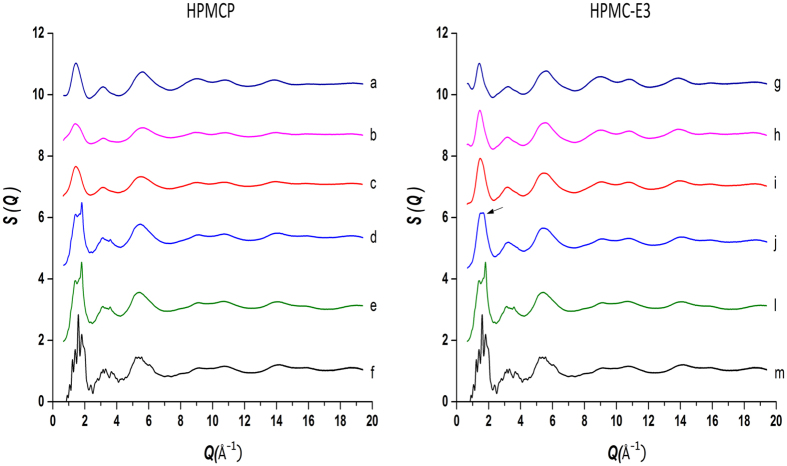
Comparison of measurement X-ray factors for spray-dried lapatinib free-base (LP), polymers and their mixtures: (**a**) pure HPMCP (spray dried); (**b**) 1:3 LP:HPMCP; (**c**) 1:1 LP:HPMCP; (**d**) 3:1 LP:HPMCP; (**e**,**l**) pure amorphous LP (spray dried); (**f**,**m**) crystalline lapatinib raw material (as is, not spray dried); (**g**) pure HPMC-E3 spray dried; (**h**) 1:3 LP:HPMC-E3; (**i**) 1:1 LP:HPMC-E3; (**j**) 3:1 LP:HPMC-E3, arrow indicates residual crystallinity. The curves have been shifted for clarity.

**Figure 2:**
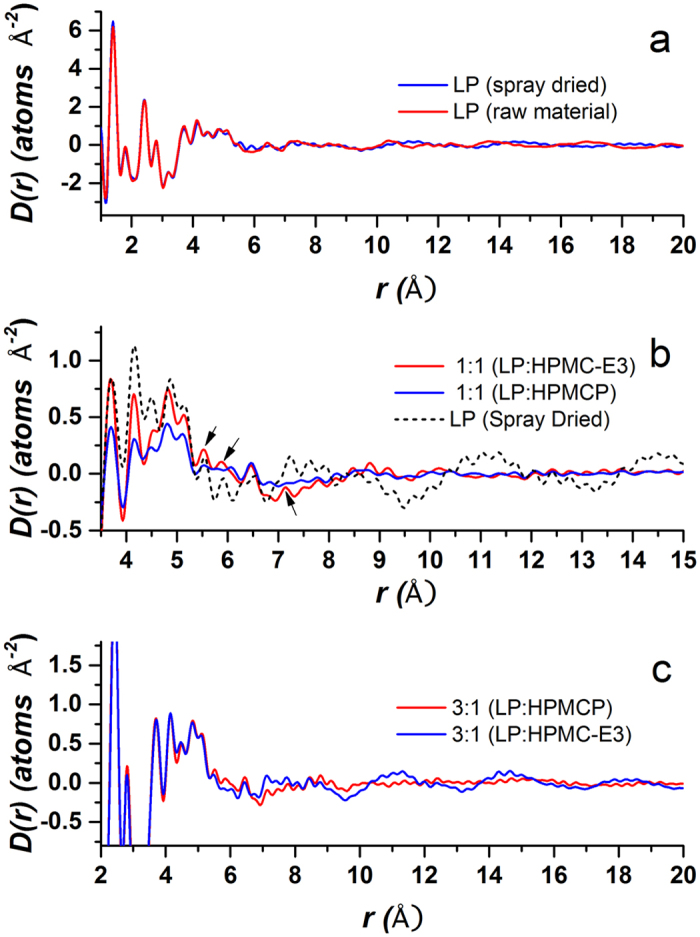
Comparison of total PDF patterns: (**a**) pure phase lapatinib samples; (**b**) 1:1 Solid dispersions of LP and polymers compared to pure spray dried from medium to long-range order. Arrows indicate examples of peaks present in both HPMC-E3-drug dispersions and pure LP (spray dried); (**c**) Overlay of 3:1 LP-polymers preparations, showing long-range order of HPMC-E3 sample.

The process of spray drying consists of the rapid evaporation of the drug solution in a hot gas stream. Spray drying is very favorable to the generation of amorphous systems because of the small droplet size and containerless environment. Exploratory studies performed using Cu K-alpha radiation indicated that all initial lapatinib-polymer powder samples were amorphous, with exception of the pure drug dispersion which immediately produced partially crystalline lapatinib. This highlights the well-recognized importance of the polymers as carrier matrices to inhibit nucleation and crystallization^27^. However, the high noise level, low resolution and lack of information present in the patterns obtained using a conventional copper-anode X-ray laboratory source made PDF and structural studies on a laboratory powder diffractometer not realiable or possible^23^,^28^. In this context the use of high-energy X-rays is fundamental to access the structural information present only at high Q ranges^23^. Figure 1 shows the comparison of measured total x-ray structure factors for LP-HPMCP and LP-HPMC-E3 mixtures, respectively. Even though all drug-polymer mixtures are XRPD amorphous, high quality x-ray structure factors and PDF curves reveal significant differences among samples. The residual crystallinity of pure phase semi-crystalline lapatinib is clearly visualized from measured X-ray structure factor for Q < 4.0 Å^−1^, given the Bragg peaks present have similar positions to the pure crystalline drug pattern (Fig. 1e,f). The PDF of the pure spray dried drug is in good agreement with the crystalline drug in the low-r range, as would be expected, as this primarily reflects the local intramolecular structure of the molecule (Fig. 2a). Differences appear as the peaks are broadened at higher-r in the range containing more intermolecular correlations, due to the amorphicity caused by the spray drying process. An intense peak at r = 1.4 Å is a result of significant contributions of carbon-carbon interactions, the most abundant in LP structure (Table 2), although carbon-nitrogen, carbon-oxygen, carbon-fluorine and oxygen-sulphur interactions all contribute to the total x-ray scattering pattern^18^.

The 3:1 LP:HPMCP structure factor (Fig. 1d) is very similar to the semicristalline LP and indicates a significant residual crystallinity that was not detected by the conventional powder diffraction. The combination of high flux of synchrotron X-rays and a 2-D area detector has been reported to be able to detect levels as low as 0.2% (w/w) of crystallinity^28^. In case of 3:1 LP:HPMC-E3 the Bragg peaks are not present in structure factor. The slight deformation in the peak at approximately Q = 1.7 Å^−1^ (arrow Fig. 1j) and PDF (Fig. 2c) shows a loss in the intermolecular order at r > 5 Å when in comparison to 3:1 LP:HPMCP, suggesting that the sample is predominantly amorphous with a possible presence of very low amount of crystalline domains. After 100 days both samples recrystallized into lapatinib free base Form 1^29^ (as well as the pure phase semi-crystalline sample), indicating that the 3:1 drug:polymer ratio samples contained enough crystalline-like interactions to eventually trigger recrystallization. Since x-ray structure factors of the 1:3 and 1:1 LP-polymer ratios do not present Bragg peaks (Fig. 1), it can be inferred that the use of amounts HPMC-E3 and HPMCP over 50% produces truly X-ray powder amorphous solid dispersions of lapatinib which can significantly influence in physical stability. This fact is in accordance with Song and co-workers who reported that a 40% drug load was suitable to obtain a stable solid dispersion of LP and HPMCP stable for 6 months at 40 °C/75%^25^. However, more structural insights are revealed when intermolecular interactions are evaluated separately.

The total measured x-ray structure factor and corresponding PDF contain intra- and inter-atom-atom correlations corresponding to drug-drug, drug-polymer and polymer-polymer interactions i.e.


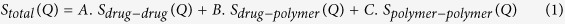


Where the drug-drug and polymer-polymer interactions have both intramolecular and intermolecular components 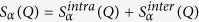 and by analogy S(Q) in reciprocal space, *S(Q),* can be replaced by *D(r)* in real space. A, B and C represent the appropriately normalized x-ray weighting factors based on the number of electrons and atomic concentrations associated with each.

In order to extract correlations from the overlapping peaks in the total PDF x-ray pattern, a series of analysis steps was performed with a view to isolating the lapatinib intermolecular drug interactions, 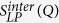, following the methodology previously outlined by Benmore^15^. Firstly, the intra- and intermolecular lapatinib functions, 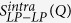 and 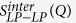
respectively, were extracted from the total structure factor of pure lapatinib using the XISF method previously described by Mou *et al*.^30^. Here, we assumed a molecular conformation corresponding to lapatinib in form 1 since amorphous samples generally crystallize into this polymorph and this is the stable form^29^. The XISF method calculates the intramolecular x-ray scattering based on the atomic x, y, z positions of the single input molecule using a zeroth order Bessel function based on a trust-region algorithim. Secondly, both the intramolecular and intermolecular polymer-polymer interactions, 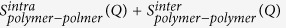
were approximated by the pattern obtained from the pure polymer structure factor. Subtracting the x-ray weighted contributions of the polymer and intramolecular LP structure factors leaves the drug interactions alone, namely 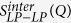 and 
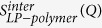
. Similarly, in real space we can write this in terms of the differential PDF as,


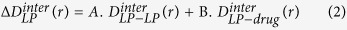


**Figure 3:**
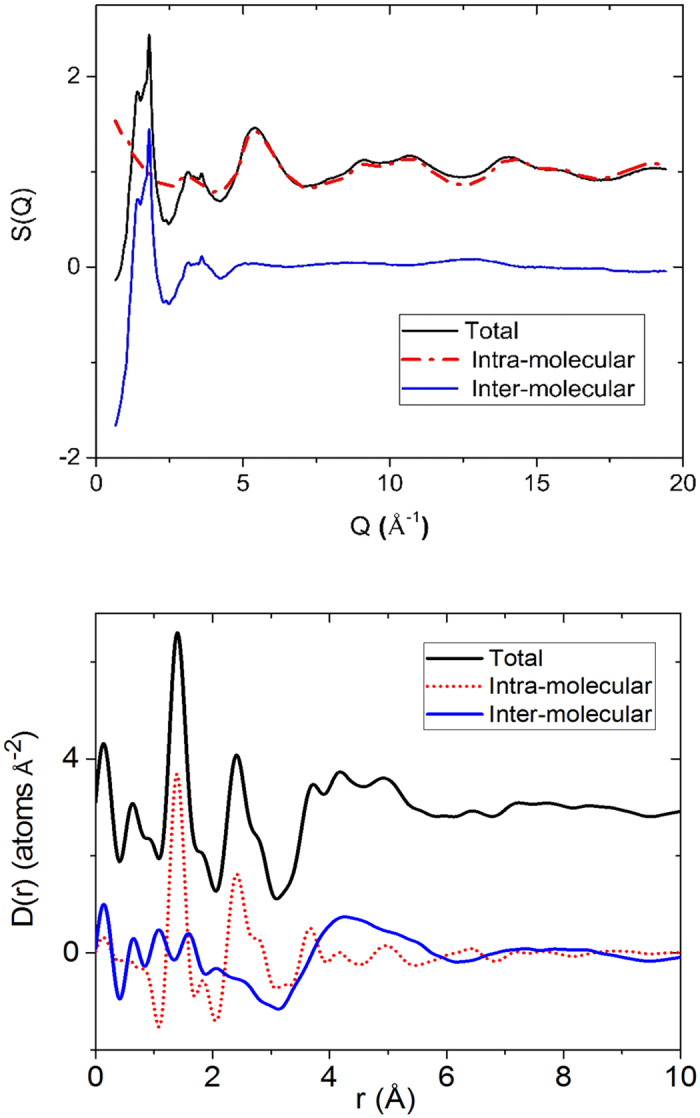
Top: The measured total x-ray structure factor for lapatinib shown along with the intramolecular fit corresponding to the scattering pattern of a single molecule, shifted for clarity. Also shown is the difference, corresponding to the intermolecular lapatinib interactions alone. Bottom: The Fourier transforms of the curves above. The total x-ray PDF above and contributions from the intramolecular and intermolecular PDF’s below.

**Figure 4:**
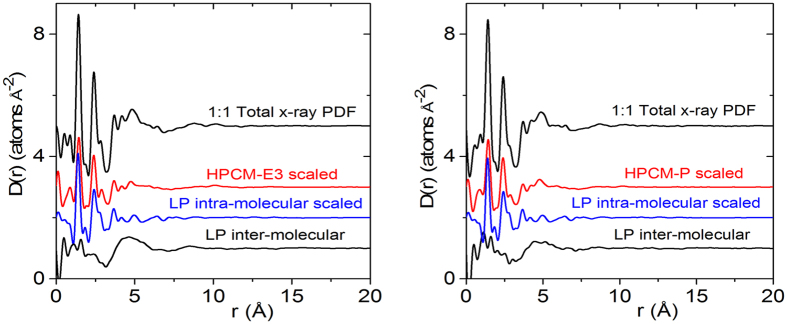
The total (measured x-ray) differential pair distribution function for the 1:1 LP:HPCM-E3 mixture (top, left) and 1:1 LP:HPCM-P mixture (top, right) each broken down into three components. Pure polymer, LP intramolecular component and LP intermolecular PDF (obtained by subtracting the polymer and LP intra from the total). The LP intermolecular PDF isolates out the intermolecular drug-drug and drug-polymer interactions present.

The function 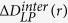 therefore represents the probability of finding an atom on drug or polymer molecule surrounding an atom on a LP molecule at the origin as a function of radial distance, r. Examples of the isolation of the intermolecular drug term for lapatinib alone and with 1:1 mixtures of HPCM-E3 and HPCM-P polymers are shown in Figs 3 and 4, respectively.

**Figure 5:**
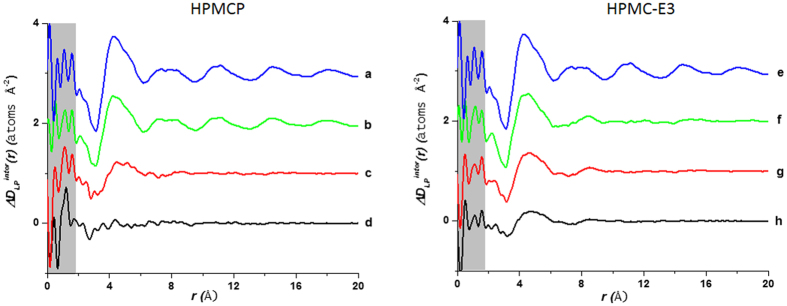
Comparison of intermolecular differential pair distribution functions of drug-polymer mixtures: (**a**,**e**) pure LP (spray dried); (**b**) 3:1 LP:HPMCP; (**c**) 1:1 LP:HPMCP; (**d**) 1:3 LP:HPMCP; (**f**) 3:1 LP:HPMC-E3; (**g**) 1:1 LP:HPMC-E3; (**h**) 1:3 LP:HPMC-E3. The gray area represents the low-r density region prone to systematic errors^15^.

The 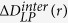 curve in Fig. 5 show well defined drug-drug interactions in pure amorphous lapatinib extending out to and beyond 20 Å. For the LP:HPMC-P system the drug rich 3:1 composition shows the similar correlations of slightly reduced magnitude indicating clusters of drug molecules on the scale of 1–2 nanometers (Fig. 5b). The 1:1 LP:HPMC-P curve shows just one broad peak around ~4.3 Å indicating orientational correlations only extend out to nearest neighbor molecules (Fig. 5c). The 1:3 curve (Fig. 5d) essentially shows a flat line implying that the LP molecules randomly dispersed in the polymer at this concentration, which supports the superior stability of those samples compared to the LP 3:1 HPMCP sample. In case of the LP:HPCM-E3 system the same analysis yields a different scenario. Here, the drug rich 3:1 composition shows a distinct first correlation at ~4.4 Å and a much weaker second peak at ~8.3 Å with nothing beyond that (Fig. 5f). The 1:1 LP:HPCM-E3 curve (Fig. 5g) and 1:3 (Fig. 5h) curves are similar to the 3:1 only showing the first and second peaks, but these are reduced in magnitude as might be expected due to the diminished drug concentration. Therefore it is suggested that at high concentrations in HPMC-E3 mixtures, lapatinib molecules do not cluster in the same way as observed in HPCM-P, but LP molecules do have distinct nearest neighbor interactions. Unfortunately it is not possible to distinguish between LP-LP and LP-polymer interactions using this method and a more detailed molecular simulation is required to deconvolute these specific molecular interactions. Nonetheless, we can use chemical arguments to help explain the observed differences.

**Figure 6:**
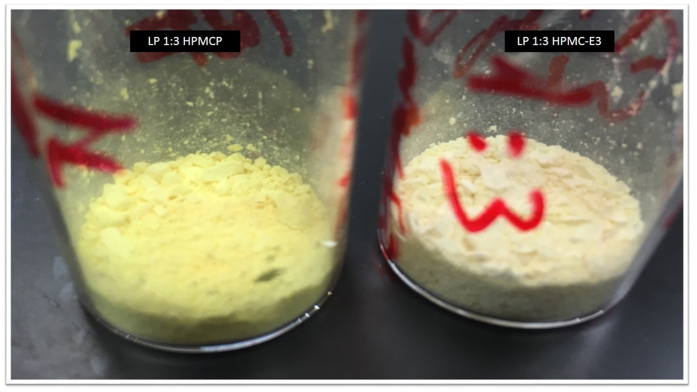
Color differences in lapatinib solid dispersions with HPMCP and HPMC-E3.

**Figure 7:**
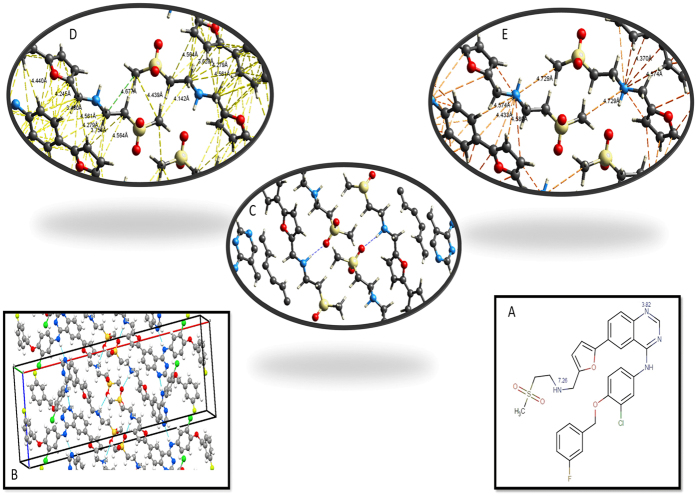
Structural aspects of Lapatinib-Lapatinib Interactions: (**A**) Molecular Structure showing pkas values for amine groups Interactions; (**B**) Unit cell of Lapatinib free base form I; (**C**) Close up of N14−H—O3 hydrogen bond; (**D**) Close up of the closest distances for Carbon-Carbon pairs; (**E**) Close up of the closest distances for Nitrogen-Carbon pairs.

**Figure 8:**
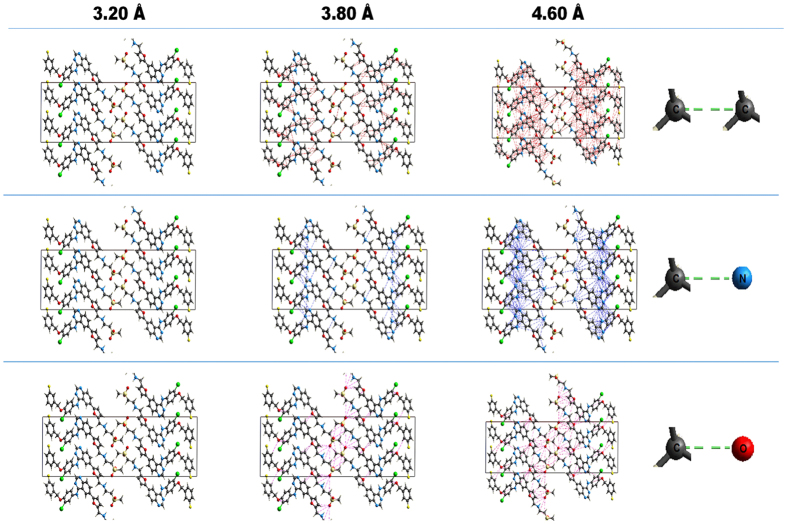
Intermolecular Carbon-carbon, Carbon-Nitrogen and Carbon-Oxygen close contacts in lapatinib unit cell^29^ ranging from 3.20 to 4.60 angstroms (view along axis b).

**Figure 9:**
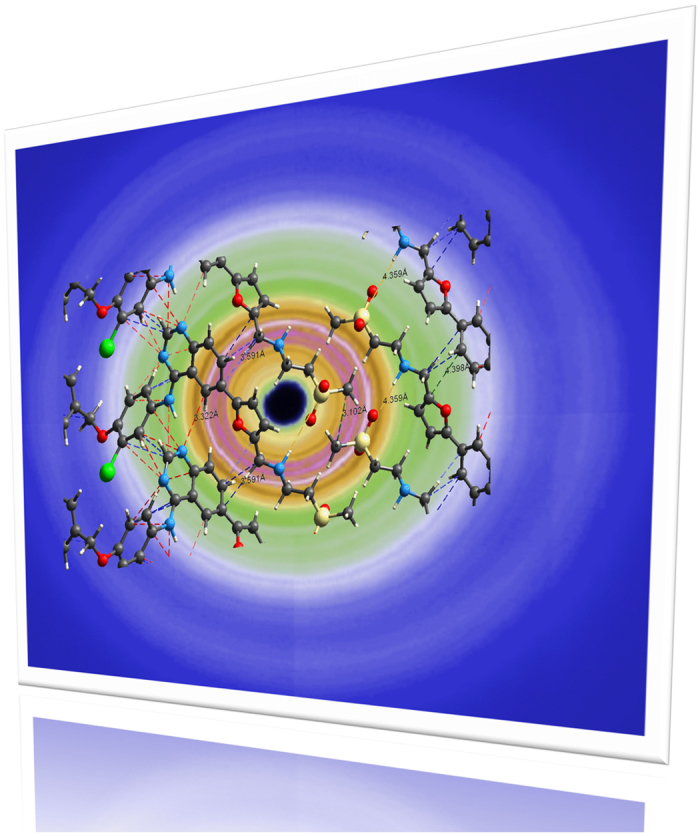
Schematic of local interactions and diffraction pattern using high-energy X-rays.

Lapatinib is well known to form ionic interactions with HPMCP through an acid-base mechanism which can be clearly observed by the differences in color of the dispersions showed in Fig. 6. Whereas HPMC-E3 dispersions exhibits a pale yellow, HPMCP samples shows as bright yellow as an evidence of salt formation^25^. This interaction is not expected in HPMC-E3, since there are no carboxylic groups to react with amines. On the other hand, lapatinib-lapatinib interactions are mainly driven by the N14−H–O3 hydrogen bond^29^ which is related to the most basic nitrogen (pka 7.26), as can be inferred from the crystal structure (Fig. 7). Also, this is the principal site for ionic pair formation with the polymer. Intermolecular differential pair distribution functions show that the first intermolecular correlation arises from a valley at 3.10 Å and reaches a peak at around 4.60 Å. This fact is consistent with the distances and the number of nearest neighbor contacts related to C-C, C-N and C-O in drug-drug interactions in the crystal, as show in Figs 7, 8 and 9. Basically, when an acid-base interaction occurs the local structure is disrupted and drug molecules are dislocated by the acidic groups of the polymer, increasing the disorder of the system. Congruently, the total D(r) for LP:HPMC-E3 closely resembles that of the pure drug (Fig. 2b; peaks are indicated by arrows) due to drug-drug contacts and 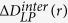 shows more peaks for LP: HPMC-E3 mixtures than for LP:HPMCP, indicating a more ordered system. Conversely, the total absence of those peaks in 1:3 LP:HPMCP preparations suggest that a complete reaction with the polymer has taken place on the N4 sites. In an analogy with the percolation rigidity theory^31^ the higher the numbers of connections and ordering, the higher the rigidity of the network will be and the more easily the system will reach the threshold for nucleation. If so, this would suggest that 1:1 LP:HPMCP and 1:3 LP:HPMC-E3 are less stable than 1:3 LP:HPMCP. In fact, this is correct. A stress test with direct exposure was carried out under 40 °C/75% RH revealing that both samples start crystallizing after 7 and 30 days, respectively, while 1:3 LP:HPMCP remains amorphous.

## Conclusion

Synchrotron X-Ray diffraction and Pair Distribution Function Analysis were successfully applied to access the local structure of an ionic drug-polymer interactions in amorphous systems. The presence of correlations extending out to beyond 20 Å were found in the total differential distribution function D(r) of pure amorphous lapatinib and also in 3:1 drug-polymer preparations indicated the presence of crystalline domains. Plus, the strategy of extracting lapatinib intermolecular drug interactions was fundamental to visualize the presence of distinct nearest neighbor for HPMC-E3 rich samples and identify that lapatinib molecules do not cluster in the same way as observed in HPCM-P. Finally, the superior physical stability of 1:3 LP:HPMCP was clarified from a structural point of view by combining the chemical knowledge, the absence of significant contacts in 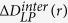 at about 3.0–6.0 Å and the pattern of nearest neighbor contacts related to C-C, C-N and C-O in drug-drug interactions. This powerful approach opens new directions in the use of structural signatures or trends at the atomic and nanometer level that could predict macroscopic behavior of amorphous solid dispersions in pharmaceutical systems.

## Methods and Materials

### Materials

Methanol (MeOH) and dichloromethane (DCM) were procured from Macron Fine Chemicals (Center Valley, PA). Lapatinib free base was purchased from LC Laboratories (Woburn, MA). HPMC-E3 (hydroxypropyl methylcellulose, Methocel^®^ E3) and HPMCP (HP-55) were obtained from Colorcon (West Point, PA, USA) and Shin-Etsu Chemical Company (Tokyo, Japan), respectively.

### Preparation of spray dried samples

Lapatinib free base and polymers were solubilized under stirring in a 70/30 (v/v) mixture of DCM/MeOH at 2% (w/v) solids concentration. Different proportions (w/w) of drug-polymer were used as follow: 1:0 (pure drug), 3:1, 1:1, 1:3 and 0:1 (pure polymer). The solutions were spray dried using a Büchi-B190 spray dryer (Büchi, Switzerland) in the following conditions: inlet temperature of 75 °C; outlet temperature of 45 °C; aspirator flow 400 (arbitrary units), pump feed rate 5 mL/min. The powder was stored in sealed glass vial and keep under refrigeration until X-ray powder diffraction (XRPD) and high-energy X-ray analysis were performed. After that the samples were stored under ambient conditions for 100 days and reanalyzed by X-ray powder diffraction (XRPD).

### Laboratory based X-ray powder diffraction

X-Ray powder diffraction patterns were measured on a Siemens/Bruker D5000 diffractometer, using Ni filtered Cu Kα radiation (λ = 1.5418 Å). The data were collected using an acceleration voltage of 40 kV and a tube current of 40 mA, step size of 0.02, step time 5 s in the angular range of 4° < 2θ < 40°

### High-Energy X-Ray Diffraction

Drug-polymer samples were mounted in polymide tubing (Cole-Parmer^®^, Vernon Hills, IL, USA) with an inside and outside diameters of 0.0710 and 0.0750 inches, respectively. The high-energy X-ray experiments were conducted on the Beamline 6-ID-D at the Advanced Photon Source, Argonne National Laboratory using a monochromatic beam of energy 100.315 keV (0.12360(5) Å) and 0.5 mm × 0.5 mm in size to minimize absorption and multiple scattering in the sample and attain high Q-values. Scattered x-rays were detected using a Perkin Elmer XRD1621 amorphous silicon area detector. The sample-detector distance of 332.9 mm was calibrated using a NIST standard CeO_2_ powder sample. The 2D scattering patterns were integrated using Fit2D^32^ and the x-ray structure factors S(Q) extracted using PDFgetX2^33^. The corresponding differential pair distribution functions D(r) were obtained by a Sine Fourier transformation using the HHS formalism as described by Keen^34^ and Susman *et al*.^18^, where,


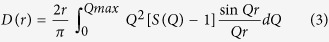


### Structure and Distance Calculations

Closest contacts distances were calculate by using CrystalExplorer 3.1 (S.K. Wolff, D.J. Grimwood, J.J. McKinnon, M.J. Turner, D. Jayatilaka, M.A. Spackman, University of Western Australia, 2012) based on crystallographic data of polymorph I^29^. Structure drawing and pKa calculation were performed using a Marvin 5.11.3 (Chemaxon, Budapest, Hungary) 2016 http://www.chemaxon.com.

## Additional Information

**How to cite this article**: de Araujo, G. L. B. *et al*. Local Structure of Ion Pair Interaction in Lapatinib Amorphous Dispersions characterized by Synchrotron X-Ray diffraction and Pair Distribution Function Analysis. *Sci. Rep.* **7**, 46367; doi: 10.1038/srep46367 (2017).

**Publisher's note:** Springer Nature remains neutral with regard to jurisdictional claims in published maps and institutional affiliations.

## Figures and Tables

**Table 1:** Examples common observations and possible interpretations of pair distribution function patterns.

Observation or event	Possible interpretation
Presence of peaks	Defined intermolecular and/or intramolecular interactions at a particular distance^9^,^13^,^14^,^15^,^35^
Presence of valleys	Absence of atoms at correspondent distance^13^,^15^,^35^,^36^
Decrease in the number of peaks and in its periodicity when comparing systems	Weakening intermolecular packing, corresponding to the loss of structural order^13^,^14^,^15^,^36^
Peaks broadening in the intermolecular range (5–20 Å)	High degree of disorder, weak intermolecular interactions^14^,^15^
Peak shifting when comparing amorphous versus crystalline materials	Changing bond lengths, conformational changes, new interactions, changes in chemical species of the system^14^,^15^
Broad and peaks or non-zero levels of intensity in non-crystalline systems	Atom-atom distances with a high degree of disorder^36^
Differences or similarities observed in experimental PDF patterns when compared to the calculated PDF.	Identification of presence or absence of nanocrystalline or amorphous domains. Useful in ruling out potential crystalline analogues and for the improvement of structural models^11^,^15^,^21^,^37^,^38^

**Table 2:** Bond search distances generated by using CrystalMaker^®^ 9.2.7 Software and calculated from single crystal structure of LP.

Bond	From (Å)	To (Å)	Mean (Å)	Number of Bonds
C-C	1.350	1.517	1.404	490
C-H	0.949	0.991	0.967	424
C-N	1.317	1.461	1.382	118
C-O	1.366	1.440	1.388	54
C-F	1.370	1.370	1.370	32
H-N	0.857	0.859	0.858	30
O-S	1.440	1.446	1.443	26
C - S	1.760	1.763	1.762	24
C-Cl	1.734	1.734	1.734	14
Cl-O	2.901	2.901	2.901	14
